# Can tree leaves be used as an alternative source of synthetic antioxidants? Use of jujube leaf extract in laying hens

**DOI:** 10.3389/fvets.2023.1305129

**Published:** 2023-12-19

**Authors:** Gözde Kılınç

**Affiliations:** Department of Food Processing, Suluova Vocational School, Amasya University, Amasya, Türkiye

**Keywords:** antioxidants, egg quality, jujube leaf, laying hens, lipid oxidation, performance

## Abstract

This study was carried out to determine the antioxidant activity of jujube (*Zizyphus jujub*a Mill.) leaf extract (JLE) and to evaluate the effects of its use as an extract in laying hen diet (Nick Brown; 32 weeks old) on performance, egg quality and lipid peroxidation. A total of 4 groups (24 replicates/group), one control (JLE-0) and three experimental groups (JLE-1, JLE-2, JLE-3), were formed and the hens were individually distributed in cages. The groups were fed with 0, 45, 90 and 135 mg/kg extract, respectively. The total phenolic content of the extract was 118.60 g gallic acid aquivalent/kg (GAE/kg) and the IC50 value was determined as 332.01 as a result of the DPPH (2, 2-diphenyl-1-picrylhydrazyl) free radical scavenging activity assay. Performance parameters except for feed conversion ratio (FCR) were not affected by the extract in the diet. Compared to the control group, FCR decreased (*p* < 0.05) and feed conversion improved in all experimental groups. The linear effect was significant for egg production (EP) (*p* < 0.05). Furthermore, egg quality parameters except for the albumen index (AI), Haugh unit (HU), shell weight (SW), and shell thickness (ST) were not affected by the extract in the diet. The highest AI and HU were in the JLE-2 group (*p* < 0.05). Besides, SW was found to increase in all experimental groups (*p* < 0.001). The highest ST was in JLE-1 (*p* < 0.001). The addition of the extract was found to slow down lipid oxidation by decreasing Thiobarbituric Acid Reactive substances (TBARs) levels on days 0 and 28 (*p* < 0.05). In conclusion, JLE can be used as a natural extract in laying hen diets.

## 1 Introduction

Some synthetic antioxidants (butylated hydroxyanisole, butylated hydroxytoluene, ethoxyquin) have been used in poultry nutrition to improve product quality (1, 2) but these products have been reported to have potential toxicological effects (3, 4). This has increased the interest in natural antioxidants as an alternative to synthetic antioxidants (5). Among these, plant-derived antioxidants have been reported to have an important place (6–8). Plants are known to contain phenolic compounds (9). It has been reported that phenolic compounds can reveal their antioxidant properties by scavenging some radical species (ROS/RNS), suppressing their (ROS/RNS) formation and protecting the antioxidant defence mechanism (10). In a study, it was reported that jujube leaves contain a good amount of phenolic substances, especially rutin and apigeni-7-glucoside (11). Jujube is a medicinal plant in the Rhamnaceae family (12). Studies (13, 14) have shown that the leaves of this plant are important as antioxidants. There are a limited number of studies on the use of *Zizyphus jujuba* leaves in animal nutrition. In various studies (15–17), the potential uses of different parts (fruit, seed meal, and powder) of the *Zizyphus jujube* in poultry (quail and broiler) were investigated. In some other studies (18–23) the use of different species of the Zizyphus genus (*Zizyphus mauritiana, Zizyphus sipina, and Zizyphus vulgaris*) in poultry (laying hens, broiler chickens, and quail) diets has been evaluated. In one of these studies (24), it was reported that the leaf extract of the *Zizyphus mauritiana* species had an immunostimulatory effect on broilers. In another study (21), it was reported that the *Zizyphus mauritiana* leaf extract (0.24 mL) added to the drinking water of quails increased the blood hemoglobin level, and it was emphasized that the extract at the level of 0.24 mL could be a good alternative source of antioxidants in quails. In another study (23), it was reported that *Zizyphus spina* leaf extract (7.5 mL kg^−1^ dose) could be used as a safe natural antioxidant to improve the reproductive status of heat-stressed chickens.

There are also different studies on the use of Zizyphus species in the nutrition of rabbits (25), fish (26), lambs (27), and goats (28).

In the literature review, there is no study investigating the effects of *Zizyphus jujuba* leaf extract on egg lipid oxidation and egg quality in laying hens. Therefore, the aim of the study was to determine the effects of three different levels of *Zizyphus jujuba* leaf extract (45, 90 and 135 mg/kg) on performance, egg quality and egg lipid peroxidation in laying hens.

## 2 Materials and methods

### 2.1 Ethical statements

This study was approved by The Ondokuz Mayıs University Animal Ethics Committee of (2018/40).

### 2.2 Collection of jujube leaves

**Figure 1:**
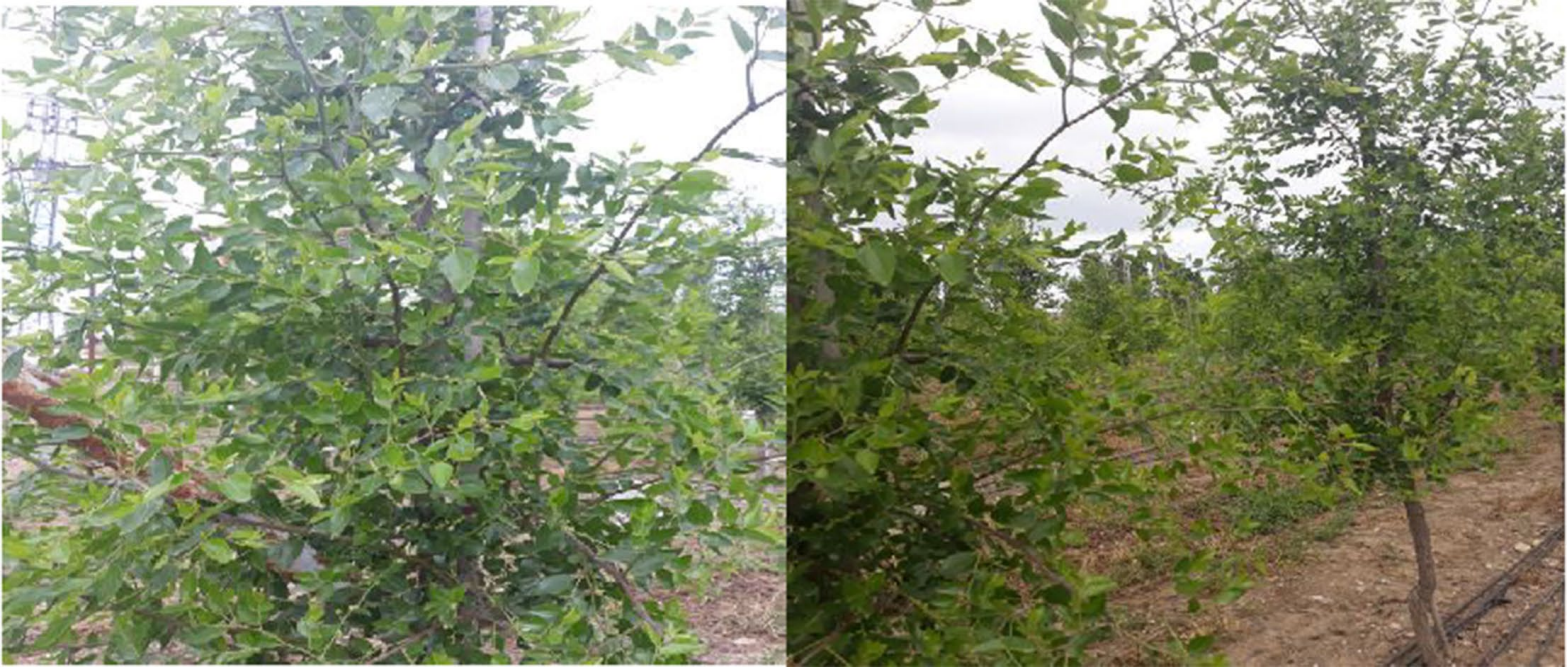
Jujube (*Zizyphus jujuba* Mill.) trees (Harmanağalı Village, Amasya, Türkiye).

Jujube (*Zizyphus jujuba* Mill.) leaves used in this study were collected from Harmanağalı Village, Suluova, Amasya Province, Türkiye in the spring (Figure 1).

### 2.3 Determination of volatile oil profile

Volatile oil was obtained by distillation method from jujube leaf powder. Volatile oil profile was performed by gas chromatography coupled to mass spectrometry (GC-MC) (Agilent: 6890 MS: 5973, New Jersey, United States). Component mass spectra were identified by comparing the retention indices of the components defined in the Flavor2, W8N05ST, and HPCH1607 libraries. Volatile components with an evaluation of less than 50% confidence were not taken into account in the library evaluation (29).

### 2.4 Preparation of jujube leaf extract

**Figure 2:**
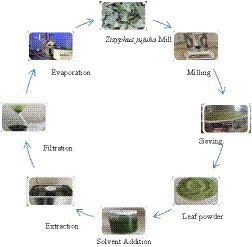
The preparation of jujube leaf extract.

The collected leaves were washed with tap water, rinsed with distilled water and left to dry in the shade. The dried leaves were ground in a laboratory blender (Waring Laboratory Blender, United States) and sieved. Extraction was performed with ethanol solution (80%) in an ultrasonic bath (Caliskan, ultrasonic cleaner, Türkiye) at a temperature (25°C) and time (30 min) determined by preliminary experiments. The resulting mixture was filtered twice through coarse filter paper. Then, the solvent was removed in a rotary evaporator at 50°C and the extracts were stored at +4°C until use (Figure 2).

### 2.5 Determination of DPPH free radical scavenging activity of the extract

DPPH (2, 2-diphenyl-1-picrylhydrazyl) free radical scavenging activity was determined using the modified method of Singh et al. (30). Accordingly, 0.01 mM DPPH solution prepared with methanol was added to solutions prepared from the extract and mixed with a vortex (IKA vortex 4 basic) for 15 s. The samples were kept in the dark for 60 min. At the end of this period, the absorbance of the samples was measured at 515 nm wavelength on a spectrophotometer (Genesys 10S UV–VIS, Therma Scientific, United States). The % reduction values were determined using the following formula (31). The results were given as IC50 values.


$$DPPHfreeradicalscavenningactivity\;\left(\%\right)=\left[\frac{1-AS}{AC}\right]x\;100$$

AS: Sample absorbance, AC: Control absorbance.

### 2.6 Determination of total phenolic content of the extract

The total phenolic content of the extract was determined according to the method of Singleton and Rossi (32). To 40 μL of solutions prepared from the extract, 2.4 mL distilled water, 200 μL Folin-Ciocalteau, 600 μL Na_2_CO_3_, and 760 μL distilled water were added, respectively. The tubes were kept in the dark for 2 h. At the end of this period, the absorbance of the samples was measured in a spectrophotometer (Genesys 10S UV–VIS, Therma Scientific, United States) at a wavelength of 765 nm. The results were expressed as g GAE/kg (GAE: Gallic Acid Equivalent).

### 2.7 Experimental design and diet

In the study, 4 groups were formed, one as control (JLE-0) and the other three as experimental groups (JLE-1, JLE-2, JLE-3), and a total of 96 chickens (Nick Brown, 32 weeks old), 24 chickens in each group, were individually distributed in 4-storey cages. G*Power 3.1. statistical program and “F tests-ANOVA Fixed effects, one-way” module were used to determine the sample size (number of chickens). The study by Radwan-Nadia et al. (33) was used as a reference for the calculation of the effect size.

The homogeneity of the chickens in terms of body weight (BW) was tested before they were placed in the cages (*p* > 0.05). An adaptation period of 1 week was applied before the start of the experiment. During the 10-week experiment, feed and water were given as *ad-libitum* and a photoperiod of 16 h of light and 8 h of dark was applied daily. Moreover, the temperature was controlled with a mini temperature data logger (datalogger; testo 174 T, Germany).

The basal diet used in the experiment was obtained from a private feed mill (laying cage hens 1st period). The nutrient composition of the diet was determined according to the method reported in AOAC (34). The content and chemical composition of the diet are given in Table 1.

**Table 1:** Chemical composition of basal diet used in the experiment.

Ingredients	kg/ton	Chemical composition	%
Maize	325.00	Dry matter	89.19
Wheat	170.576	Ether extract	4.68
Triticale	100.00	Crude fibre	5.22
Full-fat soybean (34%)	115.474	Crude protein	17.50
Sunflower meal (34%)	92.11	Crude ash	12.60
Soybean meal (46%)	20.00	D-Lysine	0.650
Hazelnut meal (42%)	28.45	D-Methionine	0.271
Corn gluten (60%)	34.04	Calcium	3.650
Vegetable oil	9.00	Phosphorus	0.375
Limestone	88.03	Sodium	0.150
Dicalcium phosphate	8.47		
Salt	3.16		
Lysine sulphate	2.10		
Premix**	2.40		
Toxin binder	1.20		
Nutrient content (analyzed) (%)			
Crude protein	Ether extract	Crude ash	Crude fibre
17.13	4.44	13.72	5.77
Calculated ME (kcal/kg)*			
2,770			

* Metabolic Energy (ME) value was calculated according to the method developed by Carpenter and Clegg (35). **Supplied the following per 2.5 kg of diet: 10.000.000 IU Vitamin A, 3.000.000 IU Vitamin D3,25.000 mg Vitamin E, 3.000 mg Vitamin K3, 3.000 mg Vitamin B1, 6.000 mg Vitamin B2, 40.000 mg Vitamin B3, 10.000 mg Vitamin B5, 4.000 mg Vitamin B6, 20 mg Vitamin B12, 50 mg Biyotin, 300.000 mg Choline, 1.000 mg Vitamin B9, 60.000 mg Fe, 5.000 mg Cu, 80.000 mg Mn, 60.000 mg Zn, 1.500 mg I. 150 mg Se, 2.500 mg Canthaxanthin (red), 1,000 mg Beta-Apo-8 (yellow), 650.000 mg DL-Methionine, 150.000 mg Optiphos 5.000 CT, 500.000 mg Kemzym, 237.908 Sepiolite.

The extracts obtained were first added to the diet little by little to form premixes and then added to the whole diet at 45, 90 and 135 mg/kg levels.

### 2.8 Parameters determined in the *in-vivo* study

Performance parameters (initial body weight, final body weight, body weight gain, egg weight, egg production, feed intake, and feed conversion ratio) were determined every 2 weeks. For this purpose, data (number of eggs, daily feed intake) were recorded daily. Eggs and feed, which were left in front of the animals, were weighed every 2 weeks.


Bodyweightgain=Finalbodyweight−Initialbodyweight

$$Egg\;production=\frac{Numberofeggs\;per\;2\;weeks}{Numberofannimals}$$

$$Feedintake=\frac{\begin{array}{l} Amountoffeedgiven\;2\;weeks-\\ Amountoffeedremaining\;per\;2\;weeks \end{array}}{Day}$$

$$Feedconversionratio=\frac{Feedintake}{Egg\;mass}$$

Egg quality characteristics (egg weight, shape index, yolk index, albumen index, Haugh unit, shell weight, shell thickness and yolk L*, a*, b* values) were determined once every 2 weeks. For this purpose, a total of 80 eggs (20 eggs from each group) were analyzed. Egg weight and shell weight were weighed with a precision balance with a sensitivity of 0.01. Egg width and length, yolk diameter, albumen width and length were measured with an insize digital caliper. Yolk and albumen heights were measured using a tripod micrometer (Mitutoyo, 0.01 mm, Japan); shell thickness was measured using a micrometer (Mitutoyo, 0.001 mm, Japan) and yolk L*, a*, b* values were measured using a colorimeter (PCE-CSM 4).

The relevant parameters were determined using the following equations (36, 37).


$$Shapeindex=\frac{Egg\;width}{Egg\;length}x\;100$$

$$Albumenindex=\frac{Albumenheight}{\begin{array}{l} Averageofalbumen\\ lengthandalbumenwidth \end{array}}x\;100$$

$$Yolkindex=\frac{Yolkheight}{Yolkdiameter}x\;100$$

$$Haughunit=100\log\;\left[H+7.57-1.7W^{0.37}\right]$$

H = Albumen height, W = Egg weight.

Egg yolk TBARs values were determined at the end of the experiment (day 0) and in eggs collected at the end of the experiment and stored at +4°C for 28 days (day 28). A total of 48 eggs were analyzed for each storage period, 12 eggs from each group. Egg yolk TBARs were determined spectrophotometrically (Genesys 10S UV–VIS, Therma Scientific, United States) using the method reported by Kılıç and Richards (38). Accordingly, 12 mL of TCA (trichloroacetic acid) solution (7.5% TCA, 0.1% EDTA, 0.1% propyl gallate) was added to egg yolk samples (2 g) and homogenized at ultra-turrax for 20–25 s and filtered through Whatmann 1 filter paper. Three ml of the filtrate was taken into glass tubes and 3 mL of 0.02 M TBA (thiobarbituric acid) solution was added. These tubes containing the solution were kept in a water bath at 100°C for 40 min and then cooled under tap water. The tubes were centrifuged at 2000 rpm for 5 min and the absorbance values were read at 530 nm wavelength in a spectrophotometer. TBARs values were calculated using the formula below and the results were given as μmol MDA/kg egg.


$$TBARs=\left[\left(\begin{array}{l} absorbance/\\ k\left(0.06\right)\;x\;2/1000 \end{array}\right)\;x\;6.8\right]\;x\;1000/sampleweight$$

k = Value obtained from the standard curve.

### 2.9 Statistical analysis

Analysis of variance (one-way ANOVA), comparison of groups (Duncan test) and effects of increasing levels of JLE (polynomial analysis) of the data obtained from the study were performed using the SPSS 20.0 package program. The effects of groups were evaluated at *p* < 0.05 level (39).

## 3 Results

### 3.1 Characteristics of dried jujube (*Zizyphus jujuba* mill.) leaf

The results of the GC/MS analysis of essential oil isolated from the jujube leaves indicated that 24 there are compounds (Table 2). Major compounds are phytol, 2-pentadecanone, myristic acid, and alpha-damascone.

**Table 2:** Volatile oil profile of jujube (*Zizyphus jujuba* Mill.) leaves.

Volatile oils g/100 g volatile oil		Volatile oils g/100 g volatile oil	
Isoelemicine	1.3	Benzoic acid	1.0
Myristic acid	5.3	Palmitic acid	1.8
1-Naphthalenol	0.9	14-Beta-H-Pregna	0.3
6-Octen-1-ol	0,9	Pyridine-3-carboxamide	0.5
2-Pentadecanone	11.2	n-Hexadecanol	0,5
1-3-Dimethyl thiepin	0.6	Manoyl Oxide	0.6
Isophytol	0.7	6,9-Pentadecadien-1-ol	0.5
2-Ethylcycloheptanone	0.5	9,12,15-Octadecatrienoic acid	1.0
Spiro (Methylenecyclopropane)	0.5	Phytol	63.8
1-Hexadecene	0.7	1-Octadecene	0.9
Alpha-Damascone	3.9	Eicosane	1.0
Methyl Hexadecanoate	0.7	n-tetracosane	0.5

### 3.2 Antioxidant activity of the extract

DPPH free radical scavenging activity and total phenolic content of jujube leaf extract are given in Table 3.

**Table 3:** Antioxidant activity of jujube leaf extract.

Total phenolic content (g GAE /kg)	IC50
118.60	332.01

GAE, gallic acid equivalent.

The total phenolic content of the extract was 118.60 g GAE/kg and the IC50 value was 332.01 as a result of the DPPH free radical scavenging activity assay.

### 3.3 Effect of extract on performance parameters

The effect of JLE on performance parameters is given in Table 4.

**Table 4:** Effects of jujube leaf extract on performance parameters.

Parameters	Groups				SEM	*p* values		
	JLE-0	JLE-1	JLE-2	JLE-3		C	L	Q
IBW	1853.83	1830.83	1878.42	1855.48	18.83	0.854	0.757	0.998
FBW	1921.00	1962.00	1964.21	1918.38	18.89	0.729	0.974	0.258
BWG	67.17	131.17	85.79	62.79	15.44	0.385	0.673	0.162
EW	64.27	64.75	64.83	63.56	0.191	0.073	0.226	**0.022**
EP	83.60	84.90	85.52	85.73	0.315	0.073	**0.013**	0.387
FI	117.46	117.15	116.43	117.24	0.541	0.918	0.777	0.607
FCR	2.235^a^	2.149^b^	2.107^b^	2.163^ab^	0.015	**0.020**	**0.049**	**0.016**

Different superscript letters (a, b) within the same row indicate significant differences between groups (*p* < 0.05). C, combined; L, linear; Q, quadratic. JLE-0, JLE-1, JLE-2, JLE-3: groups, which were fed with the addition of 0, 45, 90 and 135 mg/kg jujube leaf extract to the diet, respectively. IBW, initial body weight, g; FBW, final body weight, g; BWG, body weight gain, g; EW, egg weight, g; EP; egg production, %; FI, feed intake, g/day/hen; FCR, feed conversion ratio, g feed/g egg. SEM, standard error of mean.The bold values are *p* < 0.05.

In the laying hen diet, the effect of JLE on initial BW, FBW, BWG, egg weight and feed intake among performance parameters was insignificant (*p* > 0.05), while its effect on FCR was significant (*p* < 0.05). Compared to the control group, FCR decreased in all experimental groups, therefore, FCR improved (*p* < 0.05). As a result of polynomial analysis, linear and quadratic effects were found to be significant in terms of FCR (*p* < 0.05). Additionally, it was determined that as the JLE dose increased, a statistically insignificant numerical increase occurred in egg production.

### 3.4 Effect of the extract on egg quality characteristics

The effect of JLE on egg quality characteristics is given (Table 5).

**Table 5:** Effects of jujube leaf extract on egg quality parameters.

Parameters	Groups				SEM	*p* values		
	JLE-0	JLE-1	JLE-2	JLE-3		C	L	Q
EW (g)	63.36	64.60	64.97	63.82	0.229	0.053	0.392	**0.009**
SI (%)	78.71	79.12	78.56	79.01	0.129	0.380	0.757	0.947
YI (%)	42.91	42.38	42.36	42.33	0.115	0.220	0.087	0.267
AI (%)	9.07^b^	9.05^b^	9.63^a^	9.15^b^	0.066	**0.004**	0.165	0.081
HU	84.86^b^	84.51^b^	86.39^a^	84.82^b^	0.258	**0.044**	0.440	0.237
SW (g)	6.68^b^	7.03^a^	6.88^a^	7.01^a^	0.029	**0.000**	**0.001**	0.048
ST (μm)	366^b^	378^a^	365^b^	373^a^	0.001	**0.000**	0.527	0.415
L*	59.63	59.48	59.89	58.32	0.265	0.162	0.137	0.180
a*	12.14	12.06	12.12	12.10	0.138	0.997	0.951	0.911
b*	41.88	41.39	42.57	41.10	0.242	0.150	0.588	0.309

Different superscript letters (a, b) within the same row indicate significant differences between groups (*p* < 0.05). JLE-0, JLE-1, JLE-2, JLE-3: groups, which were fed with the addition of 0, 45, 90 and 135 mg/kg jujube leaf extract to the diet, respectively. EW, egg weight, g; SI, shape index, %; YI, yolk index, %; AI, albumen index, %; HU, haugh unit, %; SW, shell weight, g; ST, shell thickness, μm; L*, lightness; a*, redness; b*, yellowness. SEM, standard error of mean. C, combined; L, linear; Q, quadratic.The bold values are *p* < 0.05.

While the effect of JLE on egg weight, shape index, yolk index and yolk L*, a*, b* values was insignificant (*p* > 0.05), it was significant for albumen index (*p* < 0.05), Haugh unit (*p* < 0.05), shell weight (*p* < 0.001) and shell thickness (*p* < 0.001).

The highest albumen index (9.63%) and Haugh unit (86.39%) values were observed in the JLE-2 group. Compared to the control group, shell weight was higher in all experimental groups (*p* < 0.001). Furthermore, it was determined that 45 and 135 mg/kg levels of JLE in the diet increased shell thickness (*p* < 0.001). In the polynomial analysis, the linear effect was significant in terms of shell weight (*p* < 0.05).

### 3.5 Effect of the extract on egg yolk TBARs

The effect of JLE on egg yolk TBARs value is given in Table 6.

**Table 6:** Effects of jujube leaf extract on egg yolk TBARs.

Parameters	Groups				SEM	*p* values		
	JLE-0	JLE-1	JLE-2	JLE-3		C	L	Q
TBARs (day 0)	0.179^a^	0.140^b^	0.121^b^	0.143^b^	0.0062	**0.006**	**0.013**	**0.009**
TBARs (day 28)	0.203^a^	0.157^b^	0.149^b^	0.158^b^	0.0059	**0,002**	**0,004**	**0,010**

Different superscript letters (a, b) within the same row indicate significant differences between groups (*p* < 0.05). JLE-0, JLE-1, JLE-2, JLE-3: groups, which were fed with the addition of 0, 45, 90 and 135 mg/kg jujube leaf extract to the diet, respectively. TBARs, Thiobarbituric acid reactive substances, μmol MDA/kg egg. SEM, standard error of mean. C, combined; L, linear; Q, quadratic.The bold values are *p* < 0.05.

There was a significant difference between the groups in terms of egg yolk TBARs values at both day 0 and day 28 (*p* < 0.05). It was determined that 45, 90, and 135 mg/kg JLE in laying hen diets decreased the egg yolk TBARs on days 0 and 28, thus, improved egg shelf life. Moreover, in the polynomial analysis, linear and quadratic effects were found significant (*p* < 0.05).

## 4 Discussion

It was determined that JLE added to laying hen diets at different levels significantly affected only FCR among the performance parameters (*p* < 0.05). The decrease in FCR, which is one of the important parameters of performance in laying hens, in JLE-1, JLE-2 and JLE-3 groups shows the positive effect of JLE addition. In the polynomial analysis, the linear effect was significant in terms of FCR (*p* < 0.05). It is thought that this improvement in FCR is due to the bioactive components in the jujube leaf extract. When the essential oil profile of jujube leaf was evaluated in the current study, it was determined that a large proportion of it was phytol (63.8 g/100 g). It is known that phytol is used as an antibacterial, antiviral, and anti-inflammatory agent (40). It is thought that this effects on the gut microflora due to phytol may have improved FCR.

Although the difference between the groups in terms of EP was not significant, the linear effect was significant according to the polynomial analysis. It was found remarkable that egg production increased numerically with the increasing level of JLE in the diet (*p* > 0.05).

There are a limited number of studies on the use of *Zizyphus jujuba* in poultry nutrition. Son (15) reported that *Zizyphus jujuba* seed meal (0.3%, 0.6%, 0.9%) did not affect BWG and FCR.

Basiryan et al. (16) reported that the addition of *Zizyphus jujube* powder (0.75%) to broiler diets did not affect growth performance and internal organ relative weights except liver.

In another study in broilers, Yusup et al. (22) investigated the effects of different levels (10%, 15%, 20%) of bidara (*Zizyphus spina*-christi L.) leaf extract in drinking water on production and mortality and reported that bidara leaf extract in drinking water increased body weight gain, final body weight, feed intake, and water consumption. In the same study, it was determined that this extract in drinking water improved feed conversion.

Abdulameer et al. (19) reported that hydroalcoholic *Zizyphus mauritiana* leaf extract added to broiler drinking water at different levels (3, 7, 10 mL/L) did not affect body weight gain. In the same study, it was observed that feed intake was higher in the group with a 10 mL/L level of extract added to drinking water, and FCR was lower in the group with a 3 mL/L level of extract added compared to the control group, so feed conversion was better in this group.

Egg weight, shape index, yolk index, and yolk L*, a*, b* values were not affected by JLE in diet (*p* > 0.05), while albumen index, Haugh unit, shell weight and shell thickness were affected. The highest albumen index and Haugh unit were in the JLE-2 group, while the highest shell thickness was in the JLE-1 group. Compared to the control group, shell weight was higher in all experimental groups (*p* < 0.001).

Özgan (41) in a study investigating the possibilities of using grape seed oil in laying hens, reported that they thought that β-ovomucin in the structure of albumen was protected thanks to the antioxidant properties of this oil and accordingly, albumen height could increase. Many studies (13, 14, 42, 43) have reported that jujube leaf extract has antioxidant properties. Based on this information, it can be said that JLE increased the albumen index and Haugh unit in the present study due to its antioxidant properties.

Dietary strategies are valuable options to improve nutritional value and oxidative stability of poultry products (44). The addition of JLE at different levels to the laying hen diet decreased yolk TBARs levels on day 0 and day 28 (*p* < 0.05), indicating that JLE delayed lipid oxidation. In addition, both linear and quadratic effects were significant at day 0 and day 28. This positive result is thought to be due to the antioxidant properties of jujube leaves. Volatile oil of jujube leaves contains 63.8 g/100 g phytol (Table 2). Phytol is known to have a strong antioxidant effect in preventing the formation of thiobarbituric acid reactive (45).

According to the current literature review, there is no study investigating the effect of *Zizyphus jujuba* leaf extract on egg yolk lipid oxidation levels in laying hens.

However, Cellat et al. (17) reported that *Zizyphus jujuba* fruit decreased serum and breast muscle MDA levels, hence, showed a positive effect on MDA levels in their study of quails.

In addition, in another study investigating the effects of different levels of *Zizyphus jujuba* extract (500, 1,000 ppm) on the shelf life and meatball quality during storage, Gök and Bor (46) reported that the extract reduced TBARs in meatballs.

## 5 Conclusion

In this study in which the possibilities of using JLE as a natural antioxidant in laying hen diets were investigated, it was determined that JLE had a positive effect on FCR, which is one of the performance parameters. Higher albumen index and Haugh unit and lower yolk TBARs levels in eggs obtained from hens fed with a JLE-supplemented diet compared to the control group showed that JLE contributed to the preservation of egg freshness due to its antioxidant properties. Moreover, the effect of JLE on shell weight and shell thickness, which are the external quality characteristics of eggs, was also significant. In conclusion, it is thought that JLE can be used as a natural antioxidant in laying hens, but more studies should be conducted on the subject.

## Data availability statement

The original contributions presented in the study are included in the article/supplementary material, further inquiries can be directed to the corresponding author.

## Ethics statement

This study was approved by The Ondokuz Mayıs University Animal Ethics Committee of (2018/40). The study was conducted in accordance with the local legislation and institutional requirements.

## Author contributions

The author confirms being the sole contributor of this work and has approved it for publication.
